# Evaluating liquid biopsy biomarkers for early detection of brain metastasis: A systematic review

**DOI:** 10.1093/nop/npaf032

**Published:** 2025-03-18

**Authors:** Jinyue Yu, Craig Paterson, Phillippa Davies, Jennifer C Palmer, Julian P T Higgins, Kathreena M Kurian

**Affiliations:** Cancer Research Integrative Cancer Epidemiology Programme, University of Bristol, Bristol, UK; Population Health Sciences, Bristol Medical School, University of Bristol, Bristol, UK; Cancer Research Integrative Cancer Epidemiology Programme, University of Bristol, Bristol, UK; Population Health Sciences, Bristol Medical School, University of Bristol, Bristol, UK; Cancer Research Integrative Cancer Epidemiology Programme, University of Bristol, Bristol, UK; Population Health Sciences, Bristol Medical School, University of Bristol, Bristol, UK; Cancer Research Integrative Cancer Epidemiology Programme, University of Bristol, Bristol, UK; Population Health Sciences, Bristol Medical School, University of Bristol, Bristol, UK; Cancer Research Integrative Cancer Epidemiology Programme, University of Bristol, Bristol, UK; Population Health Sciences, Bristol Medical School, University of Bristol, Bristol, UK; Brain Tumour Research Centre, Bristol Medical School, University of Bristol, Bristol, UK; Cancer Research Integrative Cancer Epidemiology Programme, University of Bristol, Bristol, UK; Population Health Sciences, Bristol Medical School, University of Bristol, Bristol, UK

**Keywords:** biomarkers, brain metastasis, early detection, liquid biopsy, systematic review

## Abstract

**Background:**

Brain metastases (BMs) are the most common intracranial malignancy in adults, contributing significantly to cancer-related morbidity and mortality. Early detection is critical for optimizing treatment and improving survival. This systematic review evaluates the diagnostic potential of liquid biopsy biomarkers for detecting BM from lung, breast, and other cancers.

**Methods:**

A comprehensive search was conducted in MEDLINE, Embase, and BIOSIS databases using keywords related to liquid biopsy, biomarkers, and BMs. Data on participant characteristics, diagnostic reference standards, types of biomarkers, primary cancer origins, and diagnostic outcomes were independently extracted. Diagnostic performance was evaluated using sensitivity, specificity, and area under the curve (AUC). Risk of bias was assessed using the QUADAS-2 tool.

**Results:**

Thirty-one studies involving 5676 participants were included, assessing biomarkers such as cfDNA, miRNAs, proteins (eg, neurofilament light [NfL], glial fibrillary acidic protein [GFAP], S100B), metabolomic profiles, and multi-marker models. NfL and GFAP emerged as the most promising biomarkers, demonstrating moderate to strong diagnostic performance across multiple cancer types. Multi-marker models combining NfL and GFAP achieved sensitivity and specificity exceeding 90%. S100B showed variable performance due to differences in study designs and thresholds. Emerging biomarkers like cfDNA and metabolomic profiles showed potential but require further validation.

**Conclusions:**

Liquid biopsy biomarkers, particularly NfL and GFAP, hold promise for non-invasive BM detection. Clinical utility may be in the initial cancer workup for localized tumor to prompt brain imaging. Future research is required to validate biomarkers in larger, diverse populations across different cancer types.

**Key Points**

1. Liquid biopsy biomarkers such as NfL and GFAP show strong potential for non-invasive early detection of brain metastases from multiple primary cancers.
2. The clinical utility of these tests may be in the initial cancer workup for localized tumor to prompt imaging for brain metastasis.

**Importance of the Study**

This systematic review underscores the potential of liquid biopsy biomarkers as transformative tools for the early diagnosis of brain metastases (BMs), the most common intracranial malignancy in adults. By analyzing 31 studies involving 5676 participants, the review identifies NfL and GFAP as key biomarkers with robust diagnostic performance across multiple cancer types. Combining these biomarkers in multi-marker models demonstrates enhanced sensitivity and specificity, which may be helpful in the initial cancer workup for localized tumor to prompt CT/MRI imaging for BM. In addition, the review highlights the challenges of standardizing biomarker thresholds and methodologies and the need for large-scale validation in diverse populations.

Brain metastases (BMs) commonly arise in breast, lung, melanoma, renal, and colorectal cancers, occurring in up to 20%-40% of patients depending on the primary cancer site.^1,2^ BMs represent a huge burden of disease for healthcare systems, being responsible for approximately 98 000-170 000 cases in the USA with increasing incidence, and there is no known cure.^1^ Early detection of BMs through novel approaches may improve longer-term survival and allow development of novel treatment strategies.^3^ While the gold standard diagnostic method currently involves imaging with computed tomography (CT) or magnetic resonance imaging (MRI) scan, followed by tissue biopsy, this only occurs in a proportion of BM patients.^2,4^ These traditional diagnostic methods are an expensive, limited healthcare resource. As a result, there is a need for more accessible diagnostic tools capable of detecting and monitoring BM at various stages of the disease.

Liquid biopsy is a promising technique in cancer diagnostics and monitoring. It offers a non-invasive approach to detecting and monitoring malignancies through the analysis of circulating tumor cells (CTCs), cell-free DNA (cfDNA), proteins, and other tumor-derived biomarkers in bodily fluids such as blood, cerebrospinal fluid (CSF), and urine.^5–8^ The primary challenges in integrating liquid biopsy into pathways for the detection of BM include a requirement to improve the low and variable sensitivity of biomarkers and establish reliable standards.^9^ At present, there is a lack of systematic research evaluating the efficacy of liquid biopsy biomarkers for the detection of BM. The aim of this systematic review is to address this gap by exploring the application of liquid biopsy biomarkers of BM from various primary cancer sites, with the ultimate goal of assessing their potential as reliable, less-invasive diagnostic tools to enhance early detection and effective monitoring in clinical practice.

## Methods

This review is part of a broader systematic review project that examines the diagnostic potential of liquid biopsy biomarkers for glioma (PROSPERO; CRD42023479231). A single, comprehensive search strategy (Supplementary Table S1) was developed to cover both glioma and BM, ensuring an inclusive and exhaustive identification of relevant studies. While the search included glioma-related terms to support the overarching project objectives, the eligibility criteria for the current review specifically focused on BMs, thereby excluding studies solely addressing gliomas without BM-specific data. This systematic review is reported in accordance with the Preferred Reporting Items for Systematic Reviews and Meta-Analyses (PRISMA) guidelines.^10^

### Study Selection

Studies were eligible for this review if they: (1) had a cohort, nested case-control, case-control, or cross-sectional design; (2) examined liquid biopsy biomarkers for the early detection or diagnosis of central nervous system (CNS) metastases originating from any primary cancer; (3) included patients of any age who were diagnosed with or suspected of having BM, diagnosed using any method; and (4) included at least 10 participants in both the target group (BM+) and the control group (BM−). No restrictions were applied to the characteristics (other than sample size) of the control patients. Studies were eligible if the target group of our review was a control group from the perspective of the study authors (eg, the target group in the study was glioma, with other brain diseases including BM acting as a control group). Both diagnostic test accuracy (DTA) studies and studies comparing biomarker levels in individuals with and without BM were eligible. Studies were included if they reported on CNS metastases but provided separate results on BM. Studies were excluded if they focused only on prognosis or survival of the BM. Review articles, protocols, editorial comments, conference abstracts, dissertations, other non-peer-reviewed articles, and retracted articles were not eligible for inclusion.

We performed a comprehensive search in the MEDLINE, Embase, and BIOSIS databases from their inception until November 2023. The search utilized Medical Subject Headings (MeSH) indexing terms and keywords related to liquid biopsy, biomarkers, and BM (the full search strategy is provided in Supplementary Table S1). The search was limited to publications in the English language, with no additional restrictions. We reviewed reference lists of the included publications to identify additional studies. A specific search for gray literature was not conducted.

After automatic deduplication, 2 reviewers from the review team (C.P., P.D., and J.Y.) independently screened the titles and abstracts of all identified records using Covidence.^11^ They examined the full text of any potentially eligible article to make final decisions on eligibility. Discrepancies were resolved by a third reviewer from the review team.

### Data Extraction

We extracted data on participant characteristics, diagnostic reference standards for the BM, types of liquid biopsy/biomarkers, primary cancer origins, index test, and research outcomes. Diagnostic performance metrics, including sensitivity, specificity, cut-off values, and area under the curve (AUC) with 95% confidence intervals (CIs), were also extracted. AUC summarized diagnostic accuracy by distinguishing between BM-positive and -negative cases. Mean or median biomarker values or comparative statistics (eg, differences in means or medians) were extracted to assess diagnostic potential when AUC was not reported.

To ensure consistency, we piloted a data extraction form before starting the process. C.P., P.D., and J.Y. independently extracted data from 8 randomly selected studies. Following a consensus meeting to resolve discrepancies, J.Y. completed the data extraction, and C.P. verified all results. For multiple studies assessing different biomarkers but using the same patient database during the same period, all outcome biomarkers were extracted and linked to the ID of the earliest published study in the data extraction form.

### Risk of Bias Assessment

We assessed risk of bias and methodological quality using the Quality Assessment of Diagnostic Accuracy Studies (QUADAS-2) tool. For articles referring to the same patient cohorts but employing different methods, bias was assessed at the article level rather than the study level. A pilot assessment was conducted on the 8 randomly selected articles by 3 reviewers independently to develop review-specific criteria. For the “reference standard” domain, the team agreed that the preferred reference standard should be a confirmed diagnosis of BM through imaging (MRI/CT) combined with histological confirmation.

### Data Synthesis

We employed a narrative synthesis approach due to the heterogeneity of the included studies in terms of study design, patient populations, biomarker types, and diagnostic methods. In cases where the original article did not provide DTA statistics but included data sufficient to derive these, we estimated sensitivity, specificity, positive predictive value (PPV) and negative predictive value (NPV) using established methods.^12^ The results are organized initially by the primary cancer of the individuals examined (ie, lung cancer or breast cancer) and subsequently by the type of liquid biopsy (ie, blood, CSF) and biomarker category (eg, cellular components, proteins).

## Results

### Screening of the Literature

**Figure 1.**
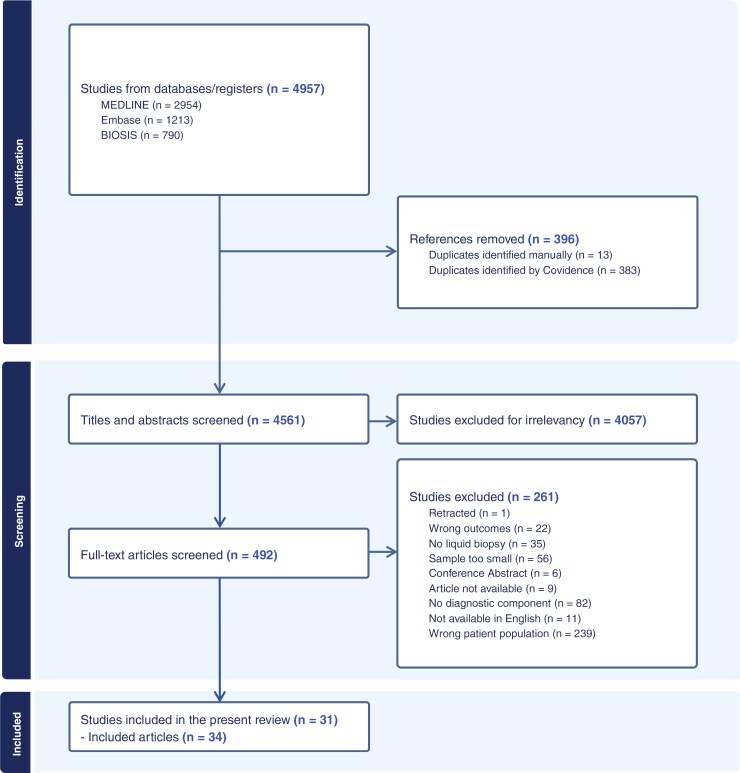
PRISMA flow chart of this systematic review.

As outlined in Figure 1, our initial literature search identified a total of 4957 records. Four hundred and ninety full-text articles were screened for eligibility. After applying the eligibility criteria, 31 studies were included in the review.

### Study Characteristics

The detailed characteristics of the included studies are summarized in Table 1. Fifteen articles focused on BM from lung cancer, 7 from breast cancer, and 9 from other single or combined primary cancers. Fourteen of the 31 studies were DTA studies. The majority of the patient population fell within the middle-aged to elderly group (45-80 years). The liquid biopsy samples analyzed were primarily derived from blood (28 studies), with a small number evaluating biomarkers in CSF (3 studies). No studies evaluated biomarkers in urine.

**Table 1.** Characteristics of the participants among included studies (*n* = 31)

Study ID	Country of the study	Types of liquid biopsy	Target group characteristics	Control group characteristics
Lung (*n* = 15)				
Cacho-Diaz 2019	Mexico	Blood (serum)	Lung cancer BM+ (SCLC = 12	Lung cancer BM− (SCLS = 9, NSCLC = 42)
			NSCLC = 136)	Age not reported
			Age mean (range) = 56.8 (21-91)	
Chen 2019	China	Blood (serum)	NSCLC BM+*n* = 100	NSCLC (stage IV) BM− *n* = 50
			Age >60 = 21 (21%)	Cerebrovascular disease = 50
			<60 = 79 (79%)	Age >60 = 11 (22%)
				<60 = 39 (78%)
Choi 2016	USA	Blood (serum)	18 Lung cancer BM+	110 Lung cancer BM−
			Age mean (SD) = 67.9 (10.5)	Age not reported
Lin 2022	China	Blood (serum)	Lung cancer BM+ *n* = 37	Control 1 Lung cancer BM− = 57
			Age: median (range) = 60 (30-79)	Control 2 HC = 64
			=60 (30-79)	Age median (range) = 62 (33-81)
Mu 2017	China	Blood (serum)	SCLC BM+*n* = 44	Control *n* = 94 (48 SCLC with other metastases
			Age mean (SD) = 66.5 (9.8)	20 SCLC no metastases
				26 underwent surgery and post-
				operative chemotherapy)
				Age mean (SD) = 67.3 (12.1)
Rojko 2020	Hungary	Whole blood	Lung cancer BM+ *n* = 352	Bone Mets: *n* = 466
			Age: <65, *n* = 197; ≥65*n* = 155	Control 1: no organ metastases *n* = 78
				Control 2: other distant organ metastasis *n* = 90
				Age:
				Bone Mets: <65 years, *n* = 216; ≥65 *n* = 250
				Control 1 <65, *n* = 37; ≥65 *n* = 41
				Control 2: <65, *n* = 42; ≥65 *n* = 48
Li 2016	USA	Blood (serum)	NSCLC (stage IV) BM+, *n* = 57 (31 had brain metastases at baseline, 26 developed brain metastases subsequently)	NSCLC BM−, *n* = 61
			Age: total participants (*n* = 118) median (range): 64 (36-85)	
			<65: *n* = 61 ≥65: *n* = 57	

Winther-Larsen 2020	Denmark	Blood (serum)	Lung cancer (stage IV) BM+, *n* = 43	Lung cancer BM− (stage I), *n* = 25
			Age: median (range) = 64 (41-85)	Age not reported
Wang 2020	China	CSF	lung adenocarcinoma BM+ (MBT), *n* = 34	Control: *n* = 129 nontumorous brain diseases NT (*n* = 36)
			Age mean (range): 57 (27-75)	primary central nervous system lymphoma (PCNSL, *n* = 59)
				secondary central nervous system involvement of systemic lymphoma (SCNSL, *n* = 11)
				lung adenocarcinoma patients without brain metastases (NMBT, *n* = 23)
				Age: 49 (17-86);
				PCNSL: 53.6 (21-76)
				SCNSL: 57.5 (20-76)
				NMBT: 53.5 (37-73)
Jin 2017	China	Whole Blood Sample (serum analyzed)	NSCLC BM+, *n* = 27;	GBM (WHO grade IV glioma) = 49
			Age >50, *n* = 23, ≤50 *n* = 4	HC = 30
				Age: >50: GBM = 37, HC = 19
				≤50: GBM = 12, HC = 11
Kondrup 2020	Denmark	Blood (serum)	NSCLC (stage IV) BM+, *n* = 22;	NSCLC (stage IV) BM−, *n* = 50
			Age: ≤65: *n* = 16	Age: ≤65: 21
			>65: *n* = 6	>65: 29
Lee 2012	South Korea	Blood (serum)	NSCLC (stage IV) BM+, *n* = 66;	NSCLC (stage IV) BM−, *n* = 161
			Age: Median (range): 66 (30-94)	Age: 66 (range 30-94)
Sert 2021	Turkey	Blood (serum)	NSCLC BM+, *n* = 56;	NSCLC (locally advanced) BM−, *n* = 152
			Age for all patients: Median (range): 63 (31-85)	
Wei 2022	China	Blood (serum)	NSCLC BM+, *n* = 204;	NSCLC BM−, *n* = 205 patients in total
			Age not reported, not significantly different between groups	(Liver metastasis *n* = 40;
				bone metastasis = 50;
				advanced NSCLC without distant organ metastasis *n* = 40;
				early-stage NSCLC, *n* = 45
				primary brain tumors, *n* = 30)
				HC = 50
Zhang 2023	China	Blood (serum)	NSCLC (TNM III and IV) BM+, *n* = 60 (III = 25, IV = 35);	NSCLC (TNM I-IV) BM−, *n* = 60 (I = 5, II = 6, III = 27, IV = 22);
			Age: ≤40 = 34;	HC = 60;
			>40 = 26	Age: BM− group:
				≤40 = 32;
				>40 = 28.
				HC:
				≤40 = 33;
				>40 = 27
Breast (*n* = 7)				
Curtaz 2022	Germany	Blood (serum)	Breast cancer BM+ *n* = 16;	Control 1: Healthy control = 67;
			Age median = 62.9	control 2 = 49 (15 breast cancer with primary cancer, 18 visceral metastases, 16 bone metastases);
				Age median
				Control = 60.3
				Primary = 61.6
				Visceral Mets = 61.1
				Bone Mets = 61.8
Darlix 2016	France	Blood (serum)	Darlix 2016 Breast cancer BM+, *n* = 88 (Grade 1/2: *n* = 34, Grade 3: *n* = 46, not reported = 8) Age <50: *n* = 28 (31.8%) 50-70: *n* = 48 (54.5%) >70: *n* = 12 (13.6%)	Breast cancer BM−, *n* = 162 (Grade 1/2: *n* = 69, Grade 3: *n* = 76, not reported = 17) Age: <50 *n* = 49 (30.2%) 50-70 *n* = 84 (51.9%) >70 *n* = 29 (17.9%)
			Darlix 2019 Breast cancer BM+ *n* = 86;	Breast cancer BM− *n* = 158;
			Age median (range) = 57.6 (26.4-79.6)	Age median (range) = 58.4 (29.6-87.2)
			Darlix 2021 Breast cancer BM+ *n* = 100 Age median (range) = 50 (33.5-62)	Breast cancer BM− *n* = 47 Age median (range) = 49.5 (33-60)
Mego 2011	USA	Whole peripheral blood	Breast cancer BM+ *n* = 22	Breast cancer BM− *n* = 270 Age not reported
			Age mean (range): CTC = 0: 55 (23-82)	
			CTC ≥1: 56 (32-81)	
Ozer 2022	Turkey	Blood (plasma)	Breast cancer BM+ *n* = 33;	Breast cancer BM− *n* = 55;
			Age: mean (SD) = 46 (9.79)	Age: mean (SD) = 46.2 (10.9)
Sato 2019	Japan	Blood (serum)	Breast cancer BM+ *n* = 51;	Breast cancer BM− *n* = 28;
			Age mean (range) = 55 (27-82)	Age: mean (range) = 49 (35-85)
Angus 2021	Netherlands	CSF	Breast cancer BM+, *n* = 11 (7 BM;	Breast cancer BM−, *n* = 122;
			4 BM and status after RT or	Age not reported
			resection);	
			Age: median = 55 (45-63)	
Bach 1989	Denmark	CSF	Bach 1989 Breast cancer BM+, *n* = 18;	Breast cancer BM−, *n* = 34;
			Age not reported	Leptomeningeal carcinomatosis = 12;
				Age not reported
			Bach 1991 Breast cancer, *n* = 23	Breast cancer BM−, *n* = 36;
			(13 parenchymal brain metastases	Age not reported
			10 leptomeningeal carcinomatosis);	
			Age not reported	
Others (*n* = 9)				
Kimura 2018	Japan	CSF	CNS+, *n* = 22 (12 Ly-CNS+, 10 CA−CNS+) Age mean (range): Ly-CNS+ 74 (58-77) Ca-CNS+ 62 (53-70)	CNS−, *n* = 18 (6 Ly-CNS−, 11 Ca-CNS−) control: non-cancer neurological disorders, *n* = 191 (INDs = 37, ANDs = 45, NINDs = 89, FNDs = 20) Age: mean (range): Ly-CNS− 53 (43-56) Ca-CNS− 74 (66-78), INDs 52 (30-63) ANDs 42 (34-54) NINDs 75 (69-78) FNDs 29 (26-42)
Bustos 2020	USA	Blood (plasma)	Cohort 1: melanoma BM+, *n* = 36;	Control 1: HC = 48.
			Age mean (SD) = 52 (28)	Age: 21-65
			Cohort 2: melanoma BM+ *n* = 24, Age mean (SD) = 60 (13)	
Faria 2018	Brazil	Blood (serum)	Adenocarcinoma BM+ (stage IV primary epithelial tumors: 19 breast, 19 lung, 2 pancreas, 11 colon, 4 prostate) Age: mean (SD): 55.2 ± 1.92	Control 1: HC = 130 Control 2: recurrent glioblastoma = 122 Age: mean (SD): HC:55.6 ± 1.68 GBM:53.4 ± 1.26
Yang 2023	China	Blood (peripheral blood)	BM+ *n* = 39; Age: mean (range) = 59 (39-78)	632 (66 patients with trigeminal neuralgia; 14 craniopharyngioma 15 ventricular meningioma 17 chordoma 93 auditory neuromas 313 meningioma 141 glioma (grade I = 1; grade II = 50; grade III = 20; grade IV = 69). Age mean (range): Trigeminal neuralgia 58.5 (19-82); Craniopharyngioma 49 (19-66) Ependymoma 47.5 (4-76) Spinal meningioma 55.5 (22-83) Acoustic neuroma 54.5 (15-83) Meningioma 53 (5-81) Glioma 48 (8-74)
Soler 2020	USA	Whole peripheral blood	BM+ *n* = 10 (lung adenocarcinoma Neuro-endocrine carcinoma Melanoma Renal cell carcinoma Metastatic urothelial carcinoma Breast adenocarcinoma Large B-cell lymphoma Basal cell adenocarcinoma of the parotid gland History of adenocarcinoma Small cell carcinoma of the lung); Age: <65 years, *n* = 15; ≥65 years *n* = 7	Control 1: HC = 10 Control 2: RN = 12 Age not reported
Twijnstra 1987	The Netherlands	CSF	Solid tumor BM+, *n* = 26 Age for total participants (*n* = 350) mean (range) 54.4 (15-92)	Control *n* = 110; Other patients *n* = 214 (23 solid tumor CMS−, 17 solid tumor epidural metastasis, 25 LM, 8 hematological tumors CNS−, 2 epidural metastasis, 9 LM, 7 meningioma, 11 malignant primary CNS tumors (glioma grade III-IV), 10 viral meningitis, 9 bacterial meningitis, 41 cerebrovascular accident, 10 polyneuropathy, 42 head injury)
Marchi 2008	USA	Blood (serum)	BM+, *n* = 14 (SVID+ *n* = 5, SVID− *n* = 9); Age not specified (mean age around 68, showing in bar chart)	SVID+ BM−, *n* = 57; SVID− BM−, *n* = 32; Age not specified (mean age around 65)
Carretero-Gonzalez 2022	Spain	Blood (plasma)	BM+, *n* = 42 NSCLC = 13 (Stage III = 2, Stage IV = 11) Breast = 14 (Stage I/II = 6, Stage III = 3, Stage IV = 5) Kidney = 4 (Stage I/II = 1, Stage IV = 3) Melanoma = 11 (Stage I/II = 2, Stage III = 4, Stage IV = 5)	Control 1: BM− *n* = 50 Lung = 12 (Stage III = 2, Stage IV = 10, NSCLC = 10, SCLC = 2) Breast = 21 (Stage I/II = 11, Stage III = 6, Stage IV = 4) Kidney = 10 (Stage I/II = 3, Stage III = 2, Stage IV = 5) Melanoma = 7 (Stage I/II = 3, Stage III = 2, Stage IV = 2) Control 2: Healthy/Cured *n* = 31 No previous cancer = 10 Breast = 9 Testicular tumor (germ cell) = 12
Kim 2021	South Korea	Blood	BM+, *n* = 70 (40 lung, 12 breast, 8 colorectal, 5 melanoma, 3 renal, 2 others) Age median (IQR) = 61 (55-69)	Control 1: BM−, *n* = 71 (37 lung, 11 breast, 10 colorectal, 6 melanoma, 4 renal, 3 others) Age: median (IQR) = 60 (55-67) Control 2: HC = 67 Age median (IQR) = 59 (53-63)
		(serum)		









Abbreviations: HC, healthy control; NSCLC, non-small cell lung cancer; SCLC, small cell lung cancer; BM+, patients with brain metastasis; BM−, patients without brain metastasis; NT, nontumorous brain diseases; PCNSL, primary central nervous system lymphoma; SCNSL, secondary central nervous system involvement of systemic lymphoma; Ly, lymphoma; CA, carcinomas; CNS+, patients with central nervous system metastasis; CNS−, patients without central nervous system metastasis; GBM, glioblastoma; RN, radiation necrosis; CSF, cerebrospinal fluid; CTC, circulating tumor cells; TNM, tumor-node-metastasis; SD, standard deviation; IQR, interquartile range.

The liquid biopsy biomarkers assessed across the studies included a variety of types, including cells and cellular components (assessed in 4 studies), genetic biomarkers such as microRNAs (miRNAs) and cfDNA (assessed in 7 studies), proteins (assessed in 20 studies), metabolites (assessed in 2 studies), and predictive models (assessed in 2 studies). The predictive models were calculated using a combination of clinical variables and biomarker levels to estimate the likelihood of BM. Moreover, the control groups varied across studies, including BM− (patients with the same primary cancer but without BMs), healthy controls (HCs), patients with other neurological or non-metastatic brain conditions (eg, glioblastoma, radiation necrosis, primary CNS lymphoma), and a combination of these. This variation in control groups highlights the heterogeneity in study designs and underscores the need for standardized approaches in future research.

### Results of Risk of Bias Assessments

The risk of bias assessments for all included studies is detailed in Supplementary Figure S1. Results from 34 articles, representing 31 studies, were assessed; risk of bias was judged to be low for patient selection in 17^13–29^; high due to inappropriate inclusion criteria or retrospective study designs in 11^30–40^; and unclear due to insufficient reporting of patient selection criteria for 6.^41–46^ For the index test, there was a low risk of bias given proper blinding and adherence to pre-specified test thresholds for 16 articles^13,15,17–24,26–28,43,45,46^; for 14, there was a high risk of bias due to the lack of blinding and use of unclear thresholds for index tests.^14,16,18,25,30,31,34–40,42^ For the remaining 4 articles, we rated risk of bias as unclear for the index test.^32,33,41,44^ Regarding the reference standard, 24 had appropriately used the preferred reference standard that was stated in the Methods section and therefore assessed as having a low risk of bias.^14,15,17–20,22,25–31,33–37,39,42,43,45,46^ A high risk of bias around the reference standard was identified in 3 articles^14,23,38^ and the remaining 8 had unclear risk of bias.^13,16,21,24,32,40,41,44^ In the flow and timing domain, risk of bias was assessed to be low in 19 articles^13,17–20,22,25–31,39,41,43–46^; and high in 11 articles due to substantial delays between the index tests and the reference standards, potentially impacting diagnostic accuracy.^14–16,21,23,24,34,35,37,38,40^ The remaining 4 had an unclear risk due to insufficient details on the timing of assessments.^32,33,36,42^

Moreover, the majority of evidence included in this review was deemed to have low applicability concerns. For patient selection, we had low concerns for 27 articles,^13–26,28,29,31–34,36–38,43–46^ high concern for 5,^27,30,35,39,40^ and unclear concerns for the remaining 2.^41,42^ For the index test, we had low concerns for 30 articles^13–17,19–29,31–35,37,38,40–46^ and high concerns for 4.^3,18,30,33,36,39,47^ Finally, concerns related to the reference standard were predominantly low; apart from 3 articles with unclear concerns,^14,21,44^ the remaining 31 articles were all deemed low concern.^13,15–20,22–43,45,46^

### Lung Cancer BMs

Supplementary Table S2 outlines the index test results for lung cancer BM+ (*n* = 15). We organize the results by types of biomarkers: cell/cellular component, miRNAs, proteins and protein-related predictive models, and metabolomic profiles.

#### Cell/cellular components (NLR, PLR, ANC, CRP, and LDH)

Studies by Rojko et al.^24^ and Sert et al.^26^ evaluated inflammatory cell biomarkers, such as neutrophil-to-lymphocyte ratio (NLR), platelet-to-lymphocyte ratio (PLR), and related indices. In Sert et al., elevated levels of NLR (≥2.6), PLR (≥198), C- reactive protein (CRP) (≥2.5 mg/dL), and lactate dehydrogenase (LDH) (≥468 IU/L) were associated with an increased risk of BMs. These biomarkers showed potential to differentiate LCBM+ from LCBM−. However, as the primary aim of the study was to evaluate BM-free survival, the analysis of predictive markers for LCBM+ was limited.

In contrast, Rojko et al. focused on comparing LCBM+ patients to those with bone metastases. Higher NLR levels in LCBM+ patients (mean value: 6.5 vs. 5.9, *P* = .004) were reported, although no evidence was found of a difference in PLR. The combined findings across these studies suggest NLR as a promising marker for differentiating between BM and other metastases, although more research is needed to refine its diagnostic accuracy. Differences in platelet count (PLT) and absolute neutrophil count (ANC) between LCBM+ and bone metastasis patients were also reported; the PLT was lower in LCBM+ patients (294 vs. 338 G/L, *P* = .001), while ANC was higher in LCBM+ patients (7.8 vs. 7.2 G/L, *P* = .010).

#### Genetic biomarkers (miR-221, miR-608, miR-504, hsa_circ_0072309, miR-100, ACKR3)

**Figure 2.**
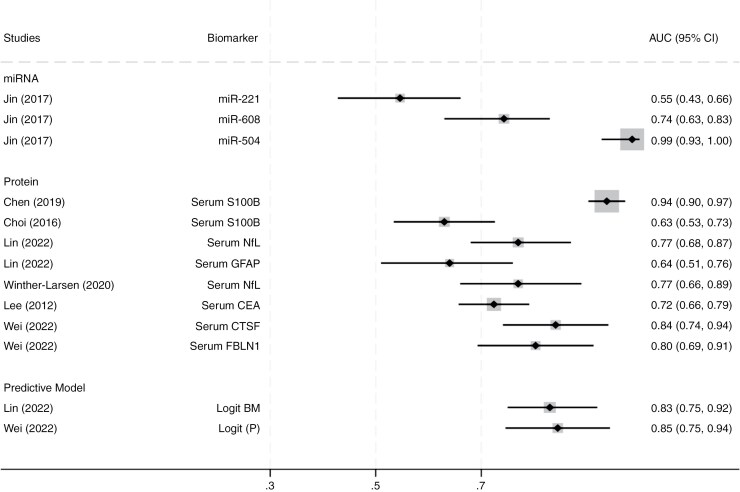
Forest plot of the diagnostic performance of biomarkers for lung cancer brain metastases (LCBM). Abbreviation: AUC, area under the receiver-operating characteristic curve. Studies are identified by author and year, with AUC values and 95% confidence intervals (CIs) indicating diagnostic accuracy for differentiating LCBM-positive and negative cases.

Jin et al.^44^ and Zhang et al.^46^ examined the diagnostic performance of circulating microRNAs (cfmiRs) in LCBM+ patients, as shown in Figure 2. While miR-504 demonstrated the highest diagnostic accuracy with an AUC of 0.99 (95% CI: 0.93-1.00), miR-608 and miR-221 showed lower diagnostic accuracy, with AUCs of 0.74 (95% CI: 0.63-0.83) and 0.55 (95% CI: 0.43-0.66), respectively. These miRNAs may play a role in identifying LCBM, but further validation is required to confirm their diagnostic utility.

Zhang et al. investigated hsa_circ_0072309, miR-100, and ACKR3 as biomarkers for LCBM+ (Supplementary Table S2). For hsa_circ_0072309, the mean expression level in BM+ patients was 1.23 (SD = 0.16), compared with 0.67 (SD = 0.15) in BM−. For miR-100, the mean expression level in BM+ was 0.89 (SD = 0.15), while it was 0.56 (SD = 0.22) in BM− patients. ACKR3 had a mean expression level of 1.14 (SD = 0.32) in BM+, compared with 0.82 (SD = 0.25) in BM−.

#### Proteins (serum S100B, NfL, GFAP, CEA, CTSF, FBLN1)

Cacho-Diaz et al.^16^ and Lee et al.^20^ investigated carcinoembryonic antigen (CEA); while Cacho-Diaz reported that a cut-off value of 15 ng/mL yielded a sensitivity of 53% and a specificity of 75% for detecting LCBM patients, Lee reported a moderate diagnostic performance of CEA (cut-off = 10, AUC = 0.724, *P* = .0001, sensitivity 76.6%, specificity 61.8%).

Chen et al.^31^ and Choi et al.^42^ evaluated the role of serum S100B. Chen et al. found serum S100B levels were associated with a lower likelihood of BM+, with an AUC of 0.94 (95% CI: 0.90-0.97). Choi et al., however, reported a lower diagnostic accuracy for S100B, with an AUC of 0.63 (95% CI: 0.53-0.73). suggesting variability in its utility across different cohorts. Mu et al. further examined serum S100B in SCLC patients and found elevated levels in BM+ compared to BM− (*P* = .001).^37^ However, in Kondrup et al., despite elevated S100B levels in LCBM+ patients, there was no statistical evidence of a difference (*P* = .852),^19^ indicating a weaker performance of S100B as a diagnostic marker in this cohort.

According to Figure 2, serum neurofilament light (NfL) was investigated in Lin et al.^36^ and Winther-Larsen et al..^40^ Both studies found consistent diagnostic value for NfL, with AUCs of 0.77 in both studies. Additionally, serum GFAP in Lin et al.^36^ were shown to be promising markers (AUC = 0.64, 95% CI: 0.51-0.76, *P* = .02). Serum CTSF and FBLN1 were evaluated by Wei et al.,^45^ who reported AUC of 0.84 (95% CI: 0.74-0.94) for CTSF and 0.80 (95% CI: 0.69-0.91) for FBLN1, respectively.

#### Predictive models (Logit BM, Logit P)

Two predictive models were developed based on serum biomarker profiles to assess the likelihood of BM in lung cancer patients. The Logit BM model in Lin et al.^36^ demonstrated a robust diagnostic capability, with an AUC of 0.83 (95% CI: 0.75-0.92. In this model, higher NfL levels, lower Karnofsky Performance Status (KPS) scores, and older age were significant contributors to BM. Similarly, the Logit P model in Wei et al.,^45^ based on serum CTSF and FBLN1 levels, showed strong diagnostic performance with an AUC of 0.845 (sensitivity: 97.7%; specificity: 69.8%), suggesting that elevated levels of these biomarkers may increase the risk of LCBM.

#### Metabolomic profiles

In addition to blood and genetic biomarkers, Wang et al.^28^ assessed CSF-based metabolomics for LCBM+ detection. They identified 27 metabolites through comprehensive CSF-based metabolomics, reporting an AUC of 0.91 (95% CI: 0.81-0.98) for distinguishing LCBM+ from lung adenocarcinoma patients without BM, indicating a good diagnostic accuracy. Moreover, the study compared LCBM+ patients with primary central nervous system lymphoma (PCNSL), secondary central nervous system involvement of systemic lymphoma (SCNSL), and nontumorous brain diseases (NT) with moderate to good diagnosis performance (0.77 for PCNSL vs. MBT, 0.87 for SCNSL vs. MBT, and 0.86 for MBT vs. NT).

### Breast Cancer BMs

Supplementary Table S3 outlines the index test results for breast cancer (BC) BM+ (*n* = 7), organized by biomarker type: cell/cellular components, miRNAs, proteins, and metabolomic profiles.

### Cell/Cellular Component Biomarkers (CTC)

Mego et al.^23^ assessed CTCs for the early detection of BCBM. CTC levels were higher in BCBM+ patients compared to BCBM− (*n* = 270). In a multiple regression analysis, CTC detection was associated with a higher likelihood of BM, with higher levels in BM+ compared with BM− patients (*P* < .001).

#### Genetic biomarkers (hsa-miR-576-3p, miR-4428, miR-4480)

Curtaz et al.^32^ and Sato et al.^25^ examined cfmiRs for the early detection of BCBM+. Curtaz et al. found that hsa-miR-576-3p had a moderate diagnostic accuracy, with an AUC of 0.67 (95% CI: 0.52-0.82). Sato et al. evaluated miR-4428 and miR-4480, with AUCs of 0.779 for miR-4428 and 0.781 for miR-4480, sensitivity and specificity as 82.4% and 64.3% for miR-4428, and 76.5% and 71.4% for miR-4480. Multiple regression results showed a difference between BM+ and BM− (OR = 2.752, 95% CI: 1.249-5.300) in miR-4428, but not for miR-4480. Since only 2 studies investigated the diagnostic performance of miRNA biomarkers, further validation is necessary to establish their diagnostic reliability.

#### Proteins (serum NSE, MMP-9, HER2 ECD, S100B, anti-S100B IgG, NfL, UCHL1, Tau, GFAP)

**Figure 3.**
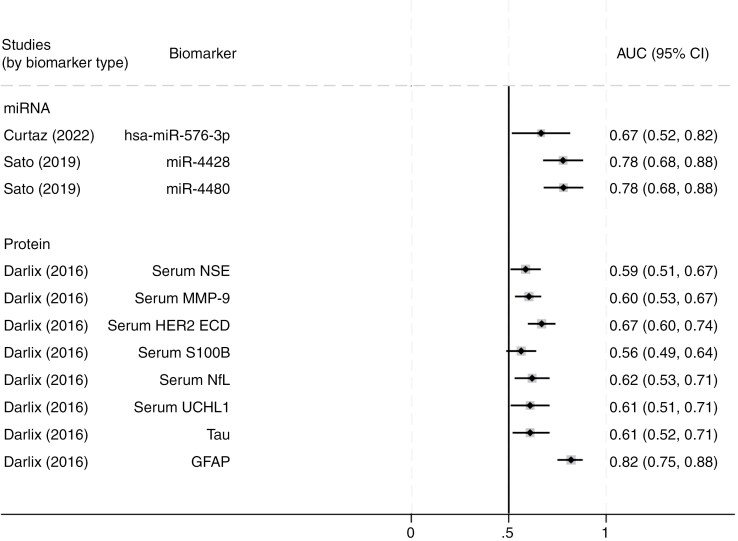
Forest plot of the diagnostic performance of biomarkers for breast cancer brain metastases (BCBM). Abbreviation: AUC, area under the receiver-operating characteristic curve. Studies are identified by author and year, with AUC values and 95% confidence intervals (CIs) indicating diagnostic accuracy for differentiating BCBM-positive and negative cases.

Darlix et al^17,33,34^ evaluated several serum proteins for their diagnostic value in breast cancer BMs. As shown in Figure 3, serum GFAP exhibited the highest diagnostic accuracy, with an AUC of 0.82 (95% CI: 0.75-0.88). Serum HER2 extracellular domain (HER2 ECD) had the second highest accuracy, with an AUC of 0.67 (95% CI: 0.60-0.74) using a cut-off value of 13.40 ng/mL. Other biomarkers, including serum NSE, MMP-9, NfL, UCHL1, and Tau, showed moderate diagnostic performance, with AUCs ranging from 0.59 to 0.62 (Supplementary Table S3; Figure 3). Serum S100B had a lower diagnostic accuracy, with an AUC of 0.56 (95% CI: 0.49-0.64).

In addition to blood-based biomarkers, CSF biomarkers were also evaluated for detecting BCBM. Angus et al.^13^ investigated the aneuploidy status of CSF-derived cfDNA using mFAST-SeqS. The study used a cut-off *z*-score of 5 to differentiate between BM+ and BM− patients. Patients with an mFAST-SeqS *z*-score ≥5 had a hazard ratio (HR) of 3.76 (95% CI: 0.96-14.75, *P* = .058) compared with those with a *z*-score < 5; the author highlighted the potential of aneuploidy detection in CSF as a promising biomarker for BM in breast cancer patients.

Moreover, Bach et al.^14^ assessed the diagnostic performance of creatine kinase BB (CK-BB) and tissue polypeptide antigen (TPA) in CSF. CK-BB was measured using a bioluminescence assay, with a cut-off value of 0.2 U/L. Median CK-BB levels in BM+ patients were higher compared with BM− controls (0.30 vs. 0.08 U/L); yet the sensitivity and specificity were 39% and 48%, respectively, indicating CK-BB may not be a strong biomarker for BM. TPA was measured using a radioimmunometric assay, with a cut-off value of 95 U/L. The median TPA level in BM+ patients was 128 U/L, compared to 69 U/L in BM− patients; sensitivity and specificity were 69% and 83%, respectively, indicating a moderate diagnostic reliability.

#### Metabolomic profiles

Ozer et al.^38^ investigated metabolomic profiles for the early detection of BCBM+. The study employed linear support vector machine (SVM) models to classify metabolomic profiles between BCBM+ and BCBM− patients. The SVM model demonstrated 96.9% diagnostic accuracy when 15 metabolites were involved in this model (AUC = 0.994, 95% CI: 0.969-1.000), and predictive accuracy of 96.9%. Those 15 effective metabolites involved in this prediction were: D-glucosyldihydrosphingosine, beta-carotene, lithocholic acid, lanosterol, phosphoguanidinoacetate, pyridoxal, eicosanoyl-CoA, 5-aminopentanoate, cholic acid, glycerophosphocolin, heptadecanoic acid, erythrose-4-phosphate, P1,P4-bis(5′-xanthosyl) tetraphosphate, neryl pyrophosphate, and urate D-ribonucleotide. The findings suggest that metabolomic profiling may offer a novel approach to identifying BM in breast cancer, but further validation is needed.

### BM from Other Origins

Supplementary Table S4 outlines the index test results for other cancers with BM+ (*n* = 9), including melanoma, colorectal cancer, and renal cell carcinoma (RCC). We organize the results by types of biomarker: cell/cellular components, genetic biomarkers, proteins, and metabolomic profiles.

### Cell/Cellular Component Biomarkers (NLR, dNLR, PLR, LMR, PNI, SII, PIV)

Yang et al.^29^ assessed a range of inflammatory and immune-related biomarkers by comparing BM+ patients (*n* = 39) with BM− controls (*n* = 632); the BM− control included patients with trigeminal neuralgia, PCNSL, and SCNSL. The AUC for differentiating acoustic neuroma from BMs using NLR was 0.749 (95% CI: 0.6482-0.8498). When combining NLR with dNLR, the AUC was 0.7481 (95% CI: 0.6457-0.8505).

Among the comparison between BM+ and all BM− controls, the majority of results were accompanied by large *P*-values (Supplementary Table S4). Apart from those, lymphocyte-to-monocyte ratio (LMR) was lower in BM+ patients compared to craniopharyngioma (3.87 vs. 6.4) and acoustic neuroma (3.87 vs. 4.71). Systemic immune-inflammation index (SII) levels were higher in BM+ patients compared to craniopharyngioma (545 vs. 245.08). Similarly, pan-immune-inflammation value (PIV) levels were higher in BM+ patients compared to craniopharyngioma (222.2 vs. 92.82) (all *P*s < .05).

#### Genetic biomarkers (cfmiRs, cfDNA)

Bustos et al.^30^ examined cfmiRs for detecting BM in melanoma patients. The study identified 164 cfmiRs that were differentially expressed between BM+ patients (*n* = 36) and HC (*n* = 48). Of these, 131 cfmiRs were upregulated, and 33 were downregulated in BM+ patients. These findings highlight the potential of cfmiRs as diagnostic biomarkers for BM. Detailed data are provided in Supplementary Table S4.

Faria et al.^43^ evaluated circulating cfDNA in patients with adenocarcinoma BM+. The study compared BM+ patients with HC (*n* = 130) and patients with recurrent glioblastoma (GBM, *n* = 50). For distinguishing BM+ patients from HCs, cfDNA demonstrated an AUC of 0.868 (95% CI: 0.797-0.939). This study also highlights the potential of circulating cfDNA as a biomarker to differentiate BM+ patients from those with recurrent GBM, with higher cfDNA levels in BM+ patients compared with GBM (median 588 ng/mL vs. 286 ng/mL, *P* < .0001).

#### Protein biomarkers (NfL, GFAP, S100B, apolipoprotein A1, DVI, CD14+, pSTAT3, PD-L1)

Kim et al.^18^ investigated the diagnostic value of serum NfL and GFAP across multiple primary cancers, including melanoma, colorectal cancer, and renal cancer. Both markers showed elevated levels in BM+ patients. The median NfL in BM+ patients was 63.7 pg/µL compared with 13.3 pg/µL in BM− patients and 12.5 pg/µL in HC (*P* < .0001 for both comparisons). The study reported age-stratified cut-offs for NfL: <60 years = 29.9 pg/µL, 60-70 years = 44.7 pg/µL, and >70 years = 63.9 pg/µL. The diagnostic performance for NfL alone showed a sensitivity of 91% and specificity of 91%.

Similarly, serum GFAP levels were higher in BM+ patients with a median of 819.5 pg/mL compared with 154 pg/mL in BM− patients and 135 pg/mL in HC (*P* < .0001 for both comparisons). The diagnostic performance of GFAP demonstrated a sensitivity of 91% and specificity of 97%. When combining NfL and GFAP, the diagnostic performance improved, with a sensitivity of 98% and specificity of 88%.

Soler et al.^39^ investigated DVI protein levels in patients with BM from solid tumors, comparing BM+ patients with radiation necrosis (RN) and HC. The study did not provide detailed data for BM+ vs. HC. When compared with RN, the DVI was elevated in BM+ patients (11.65 vs. 0.17, *P* = .005).

Marchi et al.^22^ investigated the protein biomarkers S100B and apolipoprotein A1 for detecting BM in a cohort of BM+ patients. The study compared BM+ patients with those negative for BM− (*n* = 86) and found that S100B and apolipoprotein A1 were significantly elevated in BM+ patients. The serum S100B levels were not provided but could be estimated based on Figure 2 of the original article. Since the author did not provide specific data for the comparison, estimated S100B levels were obtained from online platform (graphreader.com), which showed higher S100B levels in BM+ patients compared to BM− patients (0.25 vs. 0.15 ng/mL). Moreover, the author performed subgroup analysis by SVID (subarachnoid vessel involvement by the tumor), which is the confounding factor in the diagnostic value of S100B. BM+ patients had higher S100B levels compared to BM− patients in both SVID+ (0.22 vs. 0.16 ng/mL, *P* > .05) and SVID− subgroups (0.27 vs. 0.12, *P* < .05). However, it should be noticed that there is considerable imbalance in sample sizes between the groups (5 vs. 58 for SVID+, 9 vs. 29 for SVID−), which could introduce the potential for selection bias, as the smaller group may not be representative of the overall population. Additionally, the small sample size of the BM+ group may lead to precision bias, resulting in reduced statistical power and less reliable, more variable outcome estimates.

Carretero-Gonzalez et al.^41^ assessed pSTAT3/STAT3 and PD-L1 levels in patients with BMs from solid tumors. PD-L1 expression was significantly higher in BM+ patients compared with BM− controls, with a median difference in expression levels, as estimated from study figures. Details of the comparison are shown in Supplementary Table S4.

#### CSF protein biomarkers (LDH, PGRN)

Twijnstra et al.^27^ examined lactate dehydrogenase (LDH) as a CSF biomarker for BM detection; a greater LDH level was observed in BM+ compared to BM− (22.6 vs. 13.8 U/L, *P* < .02). Kimura et al.^35^ assessed CSF progranulin (PGRN) levels as a biomarker for detecting BM in carcinoma patients. Using a cut-off value of 2.6 pg/dL, the PGRN demonstrated a good diagnostic performance with an AUC of 0.918 (sensitivity: 90%, specificity: 85.1%) for BM+.

## Discussion

### Summary of Main Findings

This systematic review of liquid biopsy biomarkers for the early detection of BM from lung, breast, and other cancers identified a total of 31 studies. Biomarkers investigated included cell and cellular components (eg, NLR, PLR, LMR), cfDNA, miRNAs, proteins (eg, NfL, GFAP, S100B), and metabolomic profiles.

Supplementary Table S5 summarizes the evidence identified for biomarkers that were examined across multiple cancer types. NfL demonstrated high diagnostic accuracy in lung, breast, melanoma, colorectal, and renal cancers, particularly when used in multi-marker strategies. The performance of GFAP varies by cancer type, yet its diagnostic utility is enhanced when combined with NfL,^18^ highlighting the potential of multi-biomarker models to enhance diagnostic accuracy.

While S100B was frequently evaluated,^17,19,22,31,37,42^ its diagnostic performance was inconsistent due to study populations, cancer origins, and methodological thresholds. S100B performed well in some lung cancer cohorts^31,37^ but was less diagnostic in breast cancer^17^ and melanoma^22^ metastases. This variability underscores the need for standardized biomarker thresholds and methodologies to improve consistency across studies.

Other biomarkers, such as CEA, cfDNA, and genetic/metabolomic markers, showed promise but were limited by their evaluation in single studies or narrow contexts. For instance, CEA showed moderate diagnostic potential for lung^16,20^ and breast cancer BM,^33,34^ though cut-off values varied. Genetic biomarkers and metabolomic profiles were studied in single studies for each cancer type, limiting validation and generalizability.

### Comparison of Findings with Previous Evidence

A previous review showed that NfL, GFAP, and Tau are potential brain protein biomarkers for neurological damage; such biomarkers are released into the CSF and blood proportionally to the degree of neuron and astrocyte damage in different neurological disorders, such as stroke, traumatic brain injury, neurodegenerative dementia, and Parkinson’s disease^48^; however, the levels of those biomarkers used for differentiating a range of neurological diseases and monitoring disease progression need further confirmation. Animal studies have also revealed NfL as a specific serum biomarker of axonal damage severity in rat models of chemotherapy-induced peripheral neurotoxicity.^49–51^ However, current evidence from animal models mostly focused on brain injury; there is limited direct evidence specifically investigating such biomarkers in the context of BM, suggesting an area for future preclinical research to further validate these biomarkers in metastatic models.

For S100B, preclinical studies suggest that its elevation is linked to blood-brain barrier disruption and astrocytic activation caused by metastatic tumors,^52^ supporting its role as a biomarker for BM.^52,53^ While these findings supported the potential diagnostic performance of S100B, they also highlighted the need for further validation to harmonize animal and human data.

### Importance of Multi-Biomarker Models

The present review supports the use of multi-biomarker models to improve diagnostic accuracy. Notably, models such as Logit BM^36^ and Logit P^45^ evaluated for LCBM+, which incorporate multiple biomarkers alongside clinical variables, demonstrated enhanced predictive performance with AUCs over 0.80. The combination of biomarkers like NfL and GFAP, which have shown consistent results in clinical studies, strengthens the diagnostic potential of these models.^18,47^ A potential clinical use of a liquid biopsy multi-biomarker test may be in the initial cancer workup for localized tumor, which is typically done without CNS screening. If a blood biomarker (eg, NfL, GFAP) raises suspicion for CNS disease, this could prompt a brain imaging study (eg, CT, MRI). However, both GFAP and NfL need further validation in larger trials with rigorous design.

Moreover, from current literature, a biosensor approach has been discussed which allows for the real-time detection of multiple biomarkers in various neurodegenerative disease.^48^ This combined detection system of brain protein biomarkers holds significant promise for developing more specific and accurate clinical tools that can identify the type and stage of human neurological diseases with greater precision. Unfortunately, there are limited supporting evidence regarding this approach and the clinical application of diagnostic models; however, if they are validated in future prospective cohorts, these models could represent a valuable diagnostic adjunct for clinicians managing BM patients.

### Strengths and Limitations

This systematic review, to the best of our knowledge, is the first to comprehensively investigate multiple types of biomarkers for BM across various cancer origins. By employing a rigorous search strategy, we ensured a broad and systematic evaluation of evidence, capturing a diverse range of 31 studies. This provides a robust foundation for understanding the diagnostic potential of liquid biopsy biomarkers and identifying research gaps.

Nevertheless, several limitations stem from the original studies. Many studies had small sample sizes, limiting statistical power and increasing variability in results. Retrospective designs, as highlighted in Supplementary Figure S1 (QUADAS), introduced potential biases that may have influenced biomarker performance. Variability in biomarker thresholds and reporting standards further hindered comparisons; for instance, the diagnostic performance of S100B varied widely due to inconsistent cut-off values. Additionally, some studies failed to report key diagnostic metrics, such as sensitivity and specificity, limiting the ability to assess biomarkers’ diagnostic accuracy comprehensively.

### Future Directions

Future research should focus on validating biomarkers in larger, more diverse populations across different cancer types. Standardizing biomarker thresholds and methodologies, such as sample type (blood vs. CSF), is essential to improve study comparability. Multi-center trials are needed to evaluate biomarkers’ real-world clinical performance. Additionally, integrating liquid biopsy biomarkers with other diagnostic modalities, such as imaging, could enhance diagnostic algorithms. Multi-marker models combining liquid biopsy biomarkers show promise and warrant further investigation to improve diagnostic accuracy. Furthermore, we would expect to see future studies evaluating circulating tumor DNA (ctDNA), another promising biomarker^54^ that may ultimately provide a superior diagnostic tool to those evaluated in the studies we identified. Several of these approaches could advance early detection, monitoring, and treatment of BMs across cancer types.

## Supplementary material

Supplementary material is available online at *Neuro-Oncology Practice* (https://academic.oup.com/nop/).

npaf032_suppl_Supplementary_Materials

## Data Availability

This study is a systematic review and did not involve the generation of new data. All data analyzed in this review are publicly available in the studies cited throughout the manuscript. No new data were generated or analyzed in support of this research.
