# Supplementary material for: Evaluating liquid biopsy biomarkers for early detection of brain metastasis: A systematic review
Source: Neurooncol Pract. 2025 Mar 18;12(4):600–17. doi: 10.1093/nop/npaf032 (PMC12349767; doi:10.1093/nop/npaf032)
Supplement: npaf032_suppl_Supplementary_Materials [file npaf032_suppl_supplementary_materials.docx]

**Supplementary materials**

**Table S1.** Search Strategy of the original glioma systematic review

| 1 | [**Gliomas or Brain/CNS Metastases**] |
| --- | --- |
| 2 | exp Glioma |
| 3 | (glioma* or astrocytoma* or astroblastoma* or ependymoma* or subependymoma* or craniopharyngioma* or oligodendroglioma* or glioblastoma* or GBM* or oligoastrocytoma* or xanthoastrocytoma* or ganglioglioma* or gangliocytoma* or gliosarcoma* or glioneuronal* or (glial adj2 (tumor* or tumour* or cancer* or neoplasm*))).mp. |
| 4 | (brain adj1 metasta*).tw,kf. |
| 5 | ((cereb* or intracereb* or choroid plexus or crani* or intracrani* or intra-crani* or infratentori* or infra-tentori* or subtentori* or sub-tentori* or supratentori* or supra-tentori* or hypothalam* or pituitar*) adj3 metasta*).tw,kf. |
| 6 | ((mening* or leptomening*) adj3 metasta*).tw,kf. |
| 7 | ((CNS or central nervous system or spinal cord or epidur*) adj3 metasta*).tw,kf. |
| 8 | exp *central nervous system neoplasms/ or exp *brain neoplasms/ |
| 9 | or/2-8 |
| 10 | [**Biomarkers-1**] [Text-word search to find diagnostic/predictive biomarkers where these terms are discussed within three words of each other (i.e. same sentence) – precision maximising search. It helps to retrieve records which have not been fully indexed yet (i.e. records with no Subject Headings (MeSH))] |
| 11 | ((detect* or diagnos* or monitor* or predict* or prognos* or screen*) adj3 (biomarker* or bio* marker*)).tw,kf. |
| 12 | ((detect* or diagnos* or monitor* or predict* or prognos* or screen*) adj3 (tumor* or tumour* or cancer* or neoplas* or metasta*) adj3 (biomarker* or marker* or indicator*)).tw,kf. |
| 13 | 11 or 12 |
| 14 | 9 and 13 |
| 15 | ((detect* or diagnos* or monitor* or predict* or prognos* or screen*) adj3 (glioma* or astrocytoma* or astroblastoma* or ependymoma* or subependymoma* or craniopharyngioma* or oligodendroglioma* or glioblastoma* or GBM* or oligoastrocytoma* or xanthoastrocytoma* or ganglioglioma* or gangliocytoma* or gliosarcoma* or glioneuronal* or glial tumor* or glial tumour* or glial cancer* or glial neoplasm*) adj3 (biomarker* or marker* or indicator*)).tw,kf. |
| 16 | ((detect* or diagnos* or monitor* or predict* or prognos* or screen*) adj5 (brain or cereb* or intracereb* or choroid plexus or crani* or intracrani* or intra-crani* or infratentori* or infra-tentori* or subtentori* or sub-tentori* or supratentori* or supra-tentori* or hypothalam* or pituitar* or mening* or leptomening* or CNS or central nervous system or spinal cord or epidur*) adj5 (tumor* or tumour* or cancer* or neoplas* or metasta*) adj5 (biomarker* or marker* or indicator*)).tw,kf. |
| 17 | 14 or 15 or 16 |
| 18 | [**Liquid Biopsy**] |
| 19 | (liquid bio* or bod* fluid* or blood or sera or serum or plasma or cerebrospinal fluid or CSF or urine or urinalys* or circulating or cell free or extracellular or extra-cellular or exosom*).mp. |
| 20 | 17 and 19 [Combine Biomarkers-1 + Gliomas/Brain-CNS metastases + Liquid Biospy] |
| 21 | exp Central Nervous System Neoplasms/ and Neoplastic Cells, Circulating/ and Biomarkers, Tumor/ |
| 22 | 20 or 21 |
| 23 | [**Biomarkers-2**] [Broader search (increased search sensitivity) using Subject Headings or Text-words. Search terms for *diagnostic/predictive* *biomarkers* do not have to appear in the same sentence as each other ] |
| 24 | BIOMARKERS, TUMOR/ |
| 25 | *BIOMARKERS/ or *BIOLOGICAL MARKER/ |
| 26 | (biomarker* or bio* marker*).ti,kf. |
| 27 | (biomarker* or bio* marker*).ab. /freq=3 |
| 28 | or/24-27 |
| 29 | 9 and 28 |
| 30 | ((glioma* or astrocytoma* or astroblastoma* or ependymoma* or subependymoma* or craniopharyngioma* or oligodendroglioma* or glioblastoma* or GBM* or oligoastrocytoma* or xanthoastrocytoma* or ganglioglioma* or gangliocytoma* or gliosarcoma* or glioneuronal* or glial tumor* or glial tumour* or glial cancer* or glial neoplasm*) adj3 (biomarker* or marker* or indicator*)).tw,kf. |
| 31 | ((brain or cereb* or intracereb* or choroid plexus or crani* or intracrani* or intra-crani* or infratentori* or infra-tentori* or subtentori* or sub-tentori* or supratentori* or supra-tentori* or hypothalam* or pituitar* or mening* or leptomening* or CNS or central nervous system or spinal cord or epidur*) adj5 (tumor* or tumour* or cancer* or neoplas* or metasta*) adj5 (biomarker* or marker* or indicator*)).tw,kf. |
| 32 | or/29-31 |
| 33 | 19 and 32 |
| 34 | ((cell free or circulat* or exosom* or extracellular or extra-cellular) adj3 (microRNA or micro-RNA or miRNA or DNA or protein?)).mp. |
| 35 | ((cell free or circulat* or exosom* or extracellular or extra-cellular) adj3 (biomarker* or marker*)).tw,kf. |
| 36 | 34 or 35 |
| 37 | 9 and 36 |
| 38 | 33 or 37 |
| 39 | [**Diagnostic Filter**] |
| 40 | Diagnosis/ |
| 41 | Diagnosis, Differential/ |
| 42 | "Diagnostic Techniques and Procedures"/ |
| 43 | Diagnostic Test Approval/ |
| 44 | Diagnostic Tests, Routine/ |
| 45 | Molecular Diagnostic Techniques/ |
| 46 | (diagnos* adj3 (analys* or assay* or immunoassay* or classif* or differenti* or method* or kit or kits or panel? or predict* or screen* or system* or technique* or test*)).ab. |
| 47 | diagnos*.ti,kf,hw,fx. |
| 48 | (diagnos* adj2 (performance or value?)).ab. |
| 49 | (DTA or (diagnos* adj2 accura*)).tw,kf. |
| 50 | "sensitivity and specificity"/ or "predictive value of tests"/ or roc curve/ or signal-to-noise ratio/ or "limit of detection"/ |
| 51 | (sensitivity or specificity).tw,kf. |
| 52 | likelihood ratio*.tw,kf. |
| 53 | (predict* adj4 val*).tw,kf. or predict*.ti. |
| 54 | (prognos* adj4 val*).tw,kf. or prognos*.ti. |
| 55 | ((re-test or retest or test-retest) adj reliability).tw,kf. |
| 56 | ((accura* or reliab* or valid*) and (analys* or assay* or immunoassay* or classif* or detect* or diagnos* or differenti* or predict* or technique* or test*)).tw,kf. |
| 57 | Validation Study/ |
| 58 | (validat* or validity).tw,kf. |
| 59 | area under curve/ |
| 60 | observer variation/ |
| 61 | (observer adj variation*).tw,kf. |
| 62 | ((degree? or rate* or rating) adj3 agreement?).tw,kf. |
| 63 | ((detect* or diagnos*) and agreement?).tw,kf. |
| 64 | Receiver Operating Characteristic/ |
| 65 | (receiver operating characteristic* or ROC).tw,kf. |
| 66 | likelihood functions/ |
| 67 | diagnostic error/ or false negative result/ or false positive result/ or missed diagnosis/ or false negative reactions/ or false positive reactions/ |
| 68 | (false adj (positiv* or negativ*)).tw,kf. |
| 69 | (QUADAS* or STARD).mp. |
| 70 | laboratory diagnosis/ |
| 71 | (reference standard? or gold standard?).tw,kf. |
| 72 | (prognosis or progression or recurrence).hw. |
| 73 | (predict* adj3 (progressi* or recurren* or surviv*)).tw,kf. |
| 74 | progression free survival.mp. |
| 75 | or/40-74 |
| 76 | 38 and 75 |
| 77 | 22 or 76 |
| 78 | exp animals/ not humans/ |
| 79 | ((animal model* or mouse or mice or murine* or rat or rats or rodent* or muridae or murids or rabbit* or leporine* or leporidae or guineapig* or cavies or caviidae or hamster* or cricetidae or gerbil* or gerbillinae or cat or cats or feline* or felidae or dog or dogs or canine* or canidae or pig or pigs or piglet* or minipig* or swine* or porcine* or suidae or horse or horses or donkey or donkies or burros or asses or equine* or equidae or sheep or lamb or lambs or ovine or ovidae or goat or goats or cow or cows or cattle or bovine* or bovidae or primate* or monkey or monkeys or macaque or macaques or marmoset or marmosets) not human*).ti. |
| 80 | 78 or 79 |
| 81 | 77 not 80 |
| 82 | [Biomarkers in primary glioma] |
| 83 | ("21937590" or "35598381" or "32849928" or "21881830" or "31624989" or "32642707" or "29941486" or "32922940" or "19522573" or "30564636" or "24435880" or "25080476" or "29076001" or "25367878").ui. |
| 84 | 81 and 83 |
| 85 | [Biomarkers in glioma recurrence] |
| 86 | ("29797180" or "19404145" or "22492246" or "34006227" or "32038986" or "32915353" or "30719642" or "25190548" or "30413650" or "28969023" or "25785017" or "30794970" or "26171163" or "35646622" or "26230475" or "29747600" or "31611998").ui. |
| 87 | 81 and 86 |
| 88 | [Biomarkers from brain metastases] |
| 89 | ("31636790" or "34196964" or "30700265" or "33023150" or "32190309" or "34283286" or "28534372" or "26730601" or "27652205" or "18257091" or "15971200" or "34066445" or "27047250" or "29797180" or "21937590" or "31161597" or "36906568" or "34993483" or "34952628").ui. |
| 90 | 81 and 89 |

Notes: Ovid MEDLINE(R) ALL <1946 to November 01, 2023>

<https://ovidsp.ovid.com/ovidweb.cgi?T=JS&NEWS=N&PAGE=main&SHAREDSEARCHID=2pWGDlZjO5Dr43OY4iXxbFTFtvQkxFBiSPlxlKC1HKn2uCXmhkrZ6Gvm7JhuKaWy7>

**Figure S1.** Risk of bias assessment results for the 31 included studies by article (n=34) using QUADAS-2.

Table S2.Index test results of the liquid biopsy biomarkers for lung cancer brain metastasis.

| **Study ID** | **Country of the study** | **Assessed biomarker(s)** | **Test name and Manufacturer** | **Reference standard** | **Results** |
| --- | --- | --- | --- | --- | --- |
| Blood (Cell/Cellular components) | | | | | |
| Rojko 2020 | Hungary | PLT ANC ALC NLR PLR Leukocytes | Complete blood count parameters (CBC) | CT PET-CT MRI | LCBM+ vs. LC bone mets (mean):  PLT (G/L): 294 vs 338,p=0.001 ANC (G/L): 7.8 vs 7.2, p=0.010 ALC (G/L): 1.6 vs 1.7, p=0.035 NLR: 6.5 vs 5.9, p=0.004 PLR: 243 vs 270, p=ns Leukocytes (G/L):10.4 vs 9.9, p=ns |
| Sert 2021 | Turkey | PLT ANC ALC NLR PLR CRP LDH | n.r | MRI contrast-enhanced brain MRI | Elevated levels of NLR (≥ 2.6), PLR (≥ 198), CRP (≥ 2.5 mg/dL), and LDH (≥ 468 IU/L) were associated with an increased risk of brain metastases |
| Blood (Genetic biomarkers) | | | | | |
| Jin 2017 | China | miR-221 miR-608 miR-504 | Nan- oDrop 2000 (NanoDrop Technologies, TX, USA) | n.r. | miR-221:  GBM vs. LCBM+:  cut-off: ≤0.78 sensitivity=25.93%, specificity= 93.88%,  AUC= 0.546, 95% CI: 0.428-0.661   miR-608:  GBM vs. LCBM+:  cut-off: ≤0.45 sensitivity: 51.90%, specificity=97.96% AUC=0.743, 95% CI: 0.63–0.83  miR-504: GBM vs. LCBM+:  cut-off: ≤0.2 sensitivity: 88.89%, specificity: 100% AUC=0.986, 95% CI: 0.928–1.00 No related data provided for HC. |
| Zhang 2023 | China | hsa_circ_0072309 miR-100 ACKR3 | qPCR EXPRESS SYBR®- GreenER™ miRNA RT-qPCR kit (Invitrogen) | CT PET-CT MRI | Mean (SD) estimate value from Figure 2 estimated by using WebPlotDigitizer*  hsa_circ_0072309 by PCR: LCBM+ vs. LCBM-:  1.2(0.16) vs. 0.7 (0.15), p<0.001   miR-100 by PCR: LCBM+ vs. LCBM- : vs. 1.0 (0.15) vs. 0.6 (0.06), p < 0.001  ACKR3 by PCR: LCBM+ vs. LCBM-:  0.5 (0.06) vs. 0.3 (0.04), p < 0.001 |
| Blood (Protein) | | | | | |
| Cacho-Diaz 2019 | Mexico | CEA | n.r | Symptoms signs contrast-enhanced brain MRI | Serum CEA (ng/ml) cutoff =15 sensitivity=53%, specificity=75% PPV: 85%  NPV: 35% |
| Chen 2019 | China | S100B anti-S100B IgG | ELISA S100B Human ELISA Kit (Abnova, Germany) | MRI | Serum S100B (pg/ml): cut-off=13.83 AUC = 0.938, 95%CI (0.903-0.973)*, p<0.001 sensitivity = 94%, specificity = 93%  Serum anti-S100B IgG (pg/ml):  LCBM+ vs. LCBM- mean (SD): 2.70 (1.71) vs. 2.72(2.00), p=ns |
| Choi 2016 | USA | S100B anti-S100B IgG | S100B Monoclonal 2-site immunoluminometric assay - Diasorin, Stillwater, Michigan, USA Developed own ELISA for detection of S100B antibody | MRI (78%) CT(22%) | Serum S100B (ng/mL)  cut-off= 0.058 AUC = 0.63, 95%CI (0.534-0.726)* sensitivity = 89%, specificity = 43%  Anti-S100B IgG  cut-off=2.0 AU: sensitivity = 5.6%, specificity = 84.1%  Combined: anti-S100B IgG level <2.0 AU + S100B ≥0.058 ng/mL sensitivity = 89%, specificity=58.2% |
| Lin 2022 | China | NfL GFAP | Neurology 4-Plex analysis method on the Simoa platform (Quanterix; Lex- ington, Massachusetts, USA) | Symptoms signs contrast-enhanced brain MRI | Serum NfL (pg/ml) Multivariate regression: OR = 2.98, 95% CI = 1.52–5.86, p = 0.002 AUC =0.77, 95% CI = 0.68–0.87, p < 0.001.  Serum GFAP (pg/ml) AUC = 0.64, 95% CI = 0.51–0.76, p = 0.02.   Diagnostic model: Logit (BM) cut-off=0.495 AUC = 0.83 (95% CI = 0.75–0.92, p < 0.001)  Specificity:91%, sensitivity:68% Logit (BM) = 7.594 − 0.111 × (KPS) −0.052 × (age) + 1.092 × (NfL). |
| Mu 2017 | China | S100B | ELISA Wuhan Boster Biological Engineering Co., Ltd. (Wuhan, China) | Videography and pathology | Serum S100B (ng/µl):  LCBM+ : 2.138±0.174 LC other mets: 0.203±0.106 LCBM-: 0.128±0.032 HC: 0.055±0.001 F=22.87, p=0.001 by ANOVA |
| Li 2016 | USA | NSE CYFRA 21–1 Pro-GRP SCC-Ag TIMP1 HE4 | ELISA, The CYFRA 21–1 EIA (Fujirebio Diagnostics AB, Sweden). The quantitative sandwich enzyme immunoassay(Quantikine1 R&D System, Minne- apolis, Minnesota). | n.r. | Serum NSE (ng/ml): cut-off=13, OR (95%CI)=0.55 (0.15,2.07) p=0.38  Serum CYFRA 21-1 (ng/ml): cut-off=3.3, OR (95%CI)= 1.51 (0.62,3.68) p=0.37  Serum ProGRP(pg/ml): cut-off=50, OR (95%CI)=0.27 (0.03,2.42) p=0.24.  Serum SCC-Ag (ng/ml): cut-off=2.0 ng/ml, OR (95%CI)=0.41 (0.05,3.46), p=0.41 Serum HE4 (pmol/L): cut-off=65, OR (95%CI)=0.78 (0.29,2.06), p=0.61 |
| Winther-Larsen 2020 | Denmark | NfL | NF-light® assay ultra-sensitive Simoa™ HD-1 platform (Quanterix©, Lexington, MA, USA)) | CT MRI | Serum NfL (pg/mL) cut-off=24 AUC=0.77, 95% CI: 0.66-0.89 Sensitivity 69% (95%CI: 52–83%), specificity 76% (95%CI: 55–91%),  PPV 79% (95%CI:62–91%) NPV 60% (95%CI: 41–77%) |
| Kondrup 2020 | Denmark | S100B | Elecsys S100 Immunoassay (Roche Diagnostics GmbH) | CT MRI | Serum S100B (µg/l) cut-off=0.12 LCBM+: mean (range) 0.049 (0.018-0.209), 95% CI: 0.032-0.067, LCBM-: mean (range) 0.044 (0.016-0.130) , 95% CI: 0.037-0.051 p=0.852 |
| Lee 2012 | South Korea | CEA  CYFRA 21-1 CA 125  CA 19-9 SCC levels | ADVIA Centaur® XP analyzer (Siemens) for CEA,  CYFRA 21-1 immunoradiometric assay kit (Cisbio) for CYFRA 21-1,  CA 125 radioimmunoassay (RIA) kit (Immunotech) for CA 125,  CA 19-9 kit (Immunotech) for CA 19-9, and SCC RIA kit (Dinabot) for SCC | CT PET-CT MRI | Serum CEA (ng/ml) (n=216) cut-off=10  AUC = 0.724 (p=0.0001) Sensitivity: 76.6%, specificity: 61.8%.  LCBM+ vs. LCBM- Median (range):  Serum CEA (ng/ml) (n=216): 33.4 (1.5–1,000.0) vs. 5.6 (0.1–1,000.0), p<0.0001. Serum CYFRA 21-1 (ng/ml) (n=0 91):3.4 (0.9–83.3) vs.3.1 (0.5–86.2) Serum CA 19-9 (ng/ml) (n= 103): 23.0 (0.6–3,696.0) vs. 15.5 (0.1–1,857.0) Serum CA 125 (ng/ml) (n=73): 31.1 (3.3–3,879.3) vs. 25.3 (1.2–6927.0)  Serum SCC (ng/ml) (n=41): 2.0 (0.3–15.1) vs. 1.2 (0.1–560.7)  No significant difference between LCBM+ and LCBM-. |
| Wei 2022 | China | CTSF  FBLN1 | ELISA  Omin.mAbs (Alhambra, California, USA) for CTSF and FBLN1 Elabscience (Wuhan, China) for CCL20, SAA1, CXCL1, and CXCL3 Cusabio (Wuhan, China) for AXL J&L Biological (Shanghai, China) for AKR1C3 and CPNE3 | Pathology (surgical resection and/or biopsy) | Serum CTSF (U/L):  cut-off= 126.08 AUC = 0.841 sensitivity: 97.7%, specificity: 69.8%  Serum FBLN1 (U/L):  cut-off = 101.65 AUC = 0.803 sensitivity: 90.9%, specificity: 65.5%  Diagnostic model: Logit (P) cut- off =0.21 AUC = 0.845 sensitivity:97.7% specificity:69.8%  Logit(P)= 0.027*CTSF+0.048*FBLN1-8.530 |
| Cerebrospinal Fluid (Metabolomics) | | | | | |
| Wang 2020 | China | CSF-based metabolomics 27 metabolites | Comprehensive CSF-based metabolomics | Histology | LCBM+ vs. NT: (AUC=0.86, 95%CI 0.77-0.94). LCBM+ vs. PCNSL (AUC 0.77, 95% CI 0.65–0.88); LCBM+ SCNSL (AUC 0.87, 95% CI 0.72–0.99); LCBM+ vs. NMBT (AUC 0.91, 95% CI 0.81–0.98) |

Notes: n.r.=not reported, BM+=lung cancer brain metastasis, BM-=lung cancer without brain metastasis, SVID+ = Subarachnoid vessel involvement (positive), SVID− = Subarachnoid vessel involvement (negative), NT = Non-tumorous brain diseases, PCNSL = Primary central nervous system lymphoma, SCNSL = Secondary central nervous system lymphoma, NMBT = lung adenocarcinoma patients without brain metastases, HC = Healthy control, PLT = Platelet count, ANC = Absolute neutrophil count, ALC = Absolute lymphocyte count, NLR = Neutrophil-to-lymphocyte ratio, PLR = Platelet-to-lymphocyte ratio, CRP = C-reactive protein, LDH = Lactate dehydrogenase, CEA = Carcinoembryonic antigen, GFAP = Glial fibrillary acidic protein, NfL = Neurofilament light chain, ACKR3 = Atypical chemokine receptor 3, PGRN = Progranulin, DVI = HLA-Dr-Vnn2 Index, PD-L1 = Programmed death-ligand 1, OR = Odds ratio, HR = Hazard ratio, AUC = Area under the curve, PPV = Positive predictive value, NPV = Negative predictive value, ELISA = Enzyme-linked immunosorbent assay, PCR = Polymerase chain reaction, *WebPlotDigitizer: for those studies did not provide the value directly in text, an estimated value was extracted by using WebPlotDigitizer at <https://apps.automeris.io/wpd/> .

Table S3. Index test results of the liquid biopsy biomarkers for breast cancer brain metastasis.

| Study ID | Country of the study | Biomarker(s) for BM | Test name and Manufacturer | Reference standard | General description of the results |
| --- | --- | --- | --- | --- | --- |
| Blood (Cell/Cellular components) | | | | | |
| Mego 2011 | USA | CTC | The CellSearchTM system. (Veridex Corporation, Warren, NJ, USA) | Clinical signs, radiological | Multiple regression (CTC=0): BCBM+ (yes vs. no): OR(95%CI)=6.17 (2.14–17.79; p<0.001) |
| Blood (Genetic biomarkers) | | | | | |
| Curtaz 2022 | Germany | hsa-miR-576-3p hsa-miR-130a-3p | Human TaqMan Advanced miRNA Array Cards A (Thermo Fisher Scientific, Waltham, MA, USA) | n.r | BCBM+ vs all control: hsa-miR-576-3p AUC = 0.705 (95% CI: 0.566-0.844, p = 0.012)  hsa-miR-130a-3p AUC = 0.699 (95% CI: 0.582-0.816, p = 0.012)  BCBM+ vs BCBM-: hsa-miR-576-3p AUC = 0.666 (95% CI: 0.516-0.816, p = 0.048) hsa-miR-130a-3p Data not provided, estimated AUC=0.65 from Figure 6b in the original text. |
| Sato 2019 | Japan | miR-4428 miR-4480 | 3D-Gene1 microRNA Labeling kit and a 3D-Gene1 Human microRNA Oligo Chip (Toray Industries, Inc.) | CT/MRI | miR-4428: AUC=0.779  Sensitivity: 82.4%, specificity 64.3%  Multiple regression: OR (95%CI)=2.752 (1.249–5.300)  miR-4480: AUC=0.781  Sensitivity: 76.5%, specificity 71.4% Multiple regression: not significant, data not provided. |
| Blood (Protein) | | | | | |
| Darlix 2016 | France | NSE MMP-9 HER2 ECD S100B anti-S100B IgG | NSE, MMP-9 and HER2 ECD: (Human MMP-9 Quantikine Kit R&D Systems, Minneapolis, ELSA-NSE RIA kit, Cisbio assays, Gif-sur-Yvette, France and Nuclea Diagnostic Labora- tories kit, LLC for HER2 ECD); S100ß: Elecsys S100 Immunoassay (Roche Diagnostics GmbH, Mannheim, Germany). | MRI/ CT | HER2 ECD (ng/mL）:  Cut-off=13.40 AUC=0.669 (95%CI 0.598-0.740) Sensitivity=0.74, specificity=0.60.  NSE (µg/L): Cut-off=11.30 AUC=0.587 (95%CI 0.509-0.665) Sensitivity=0.49, specificity=0.72.  MMP-9 (ng/ml): Cut-off=245.77 AUC=0.604 (95%CI 0.532-0.667) Sensitivity=0.83, specificity=0.37.  S100B(ug/ml): Cut-off=0.08 AUC=0.564 (95%CI 0.487-0.641) Sensitivity=0.33, specificity=0.84. |
| Darlix 2016 (published in 2019) |  | Tau CA 15-3 CEA HER2  ECD MMP-9 | HER2 ECD - ELISA - Nuclea Diagnostic Laboratories Tau - digital ELISA using Single Molecule array (Simoa)  CA 15-3 - assay - Cisbio asssays, Gif sur Yvette, France CEA - Elecsys CEA test - Roche Diagnostics, Meylan, France MMP-9 - ELISA - Human MMP-9 Quantikine Kit, R&D systems, Minnessota, USA |  | CEA (ng/mL): Cut-off=10 HR (95%CI)=2.71 (1.10-6.68), p=0.03  CA 15-3 (U/mL): Cut-off=30 HR (95%CI)=0.33 (0.14-0.78), p=0.012  Tau (pg/mL): Cut-off=3.17 HR (95%CI)=3.98 (1.11-14.30), p=0.034  HER2-ECD (ng/mL):  Cut-off=12.7 HR (95%CI)=7.26 (2.32-22.71), p=0.001  MMP-9 (ng/mL): Cut-off=245.78 HR (95%CI)=4.69 (2.05-10.73), p<0.001 |
| Darlix 2016 (Published in 2021) |  | NfL UCHL1 Tau  GFAP CA 15-3 CEA | NfL, UCHL1, tau and GFAP: Neurology-4-plex assay Lot 501715, Simoa technology, Quanterix Corporation, Lexington, MA, USA. 15-3 (CA 15-3) (ELISA-CA15-3 Cisbio assays, Gif sur Yvette, France) and carcinoembryonic antigen (CEA) (Elecsys CEA test Roche Diagnostics, Meylan, France), |  | ROC Curves - cut off = median values in cell K9 NfL (pg/mL): Cut-off=30.3 AUC = 0.62 (95CI: 0.53-0.71) Sensitivity = 0.57, specificity = 0.64  UCHL1(pg/mL): Cut-off=10.7 AUC = 0.61 (95%Ci:0.51-0.71) Sensitivity = 0.57, specificity = 0.66  Tau (ng/mL): Cut-off=0.55 AUC = 0.61, 95%CI:0.52-0.71) Sensitivity = 0.56, specificity = 0.64  GFAP (ng/mL): Cut-off=189.6 AUC = 0.82 95%CI: 0.75-0.88) Sensitivity = 0.67, specificity= 0.87  CA15-3 (U/mL): Cut-off=30 OR (95%CI)=1.21(0.58-2.50), p=0.615  CEA (ng/mL): Cut-off: 10 OR (95%CI)=1.96 (0.87-4.42), p=0.095 |
| Blood (Metabolomics) | | | | | |
| Ozer 2022 | Turkey | Metabolomic profiles | GC–MS based metabolomics LC-qTOF-MS-based metabolomics | n.r. | Linear supported vector machines (SVM) mode of different numbers of metabolites (5, 10, 15, 25, 50 and 100) using ROC curve analysis:  Highest predictive accuracy when 15 metabolites involved in the SVM mode AUC=0.994 (95%CI: 0.969-1.000)  predictive accuracy=96.9%  The 15 effective metabolites involved in this prediction are:  D-Glucosyldihydrosphingosine, beta-caratone,  Lithocholic acid, Lanosterol,  Phosphoguanidinoacetate, Pyridoxal,  Eicosanoyl-CoA, 5-Aminopentanoate,  Cholic acid, Glycerophosphocolin,  Heptadecanoic acid, Erythrose- 4-phosphate,  P1,P4-Bis(5’-xanthosyl) tetraphosphate,  Neryl pyrophosphate, Urate D-ribonucleotide. |
| Cerebrospinal Fluid (Protein) | | | | | |
| Angus 2021 | Netherlands | Aneuploidy status in CSF-derived cfDNA | mFAST-SeqS（modified fast aneuploidy screening test-sequencing system） | MRI | Aneuploidy status (mFAST-SeqS z-score) Cut-off= 5 mFAST-SeqS z-score (<5 vs. ≥5): HR (95%CI)=3.76 (0.96–14.75), p=0.058 |
| Bach 1989 | Denmark | CK-BB | bioluminescence assay using a CK-B kit (LKB-Wallac) | CT and/or autopsy examination | CK-BB (U/L): Cut-off=0.2 BCBM+ : Median (range)=0.14 (0.04-0.83) Sensitivity= 39%, specificity 48% PPV 41% NPV 77% |
| Bach 1989 (published in 199) |  | TPpA | TPpA was measured using a radioimmunometric assay (IRMA, TPpA Prolifigen; AB Sangtec Medical, Stockholm, Sweden) | Clinical course, neurological signs, CSF cytology, and autopsy | TPpA (U/L): Cut-off=95 BCBM+ : Median (range)=148 (3-2101) Sensitivity 69%, specificity 83% PPV 53%  NPV 91% |

Notes: n.r.=not reported, BCBM+=breast cancer brain metastasis, BCBM-= breast cancer without brain metastasis, HC = Healthy control, SVID+ = Subarachnoid vessel involvement (positive), SVID− = Subarachnoid vessel involvement (negative), NT = Nontumorous brain diseases, PCNSL = Primary central nervous system lymphoma, SCNSL = Secondary central nervous system lymphoma, CTC = Circulating tumor cell, cfDNA = Cell-free DNA, NfL = Neurofilament light chain, GFAP = Glial fibrillary acidic protein, UCHL1 = Ubiquitin carboxyl-terminal hydrolase L1, CK-BB = Creatine kinase BB, TPpA = Tissue polypeptide antigen, CEA = Carcinoembryonic antigen, HER2 ECD = Human epidermal growth factor receptor 2 extracellular domain, NSE = Neuron-specific enolase, MMP-9 = Matrix metalloproteinase-9, CA 15-3 = Cancer antigen 15-3, HR = Hazard ratio, OR = Odds ratio, AUC = Area under the curve, PPV = Positive predictive value, NPV = Negative predictive value, mFAST-SeqS = Modified fast aneuploidy screening test-sequencing system, GC–MS = Gas chromatography-mass spectrometry, LC-qTOF-MS = Liquid chromatography–quadrupole time-of-flight mass spectrometry.

Table S4. Index test results of the liquid biopsy biomarkers for brain metastasis from other origins.

| Study ID | Country of the study | Which biomarker(s) tested | Test name and Manufacturer | Reference standard | General description of the results |
| --- | --- | --- | --- | --- | --- |
| Blood (Cell/Cellular components) | | | | | |
| Yang 2023 | China | NLR dNLR PLR LMR PNI SII PIV | NLR (neutrophil/lymphocyte count), dNLR ([white blood cell count – neutrophil count]/lymphocyte count), PLR (platelet count/lymphocyte count), LMR (lymphocyte count/monocyte count), PNI (albumin count + lymphocyte count ∗5), SII (platelet count ∗ neutrophil count/lymphocyte count), and PIV (neutrophil count ∗ platelet count ∗ monocyte count/lymphocyte count) | Biopsy or postoperative pathology | NLR median (range):  BM+ :3.65 (1.2–16.38) p<0.05 for: trigeminal neuralgia: 2.11 (0.97–18.39) craniopharyngioma: 1.74 (0.9–15.95) acoustic neuroma: 2.11 (0.83–4.79)  LMR median (range): BM+ : 3.87 (0.38–12.56), p<0.05 for: craniopharyngioma: 6.4 (1.05–8.59) acoustic neuroma: 4.71 (1.72–9.83)  SII median (range): BM+ :545.5 (18.97–3411.13), p<0.05 for: craniopharyngioma:245.08 (106.6–3525.48)  PIV median (range): BM+ : 222.2 (23.54–3445), p<0.05 for: craniopharyngioma: 92.82 (35.18–2115)  dNLR median (range):  BM+: 1.34 (1.09–1.97), p>0.05 for all comparisons  PLR median (range):  BM+: 127.8 (27.59–278.6), p>0.05 for all comparisons  PNI median (range):  BM+: 49.7 (33.9–58.75), p>0.05 for all comparisons  AUC (95%CI): Acoustic neuroma vs. brain metastases  NLR: 0.7490 (0.6482–0.8498) NLR + dNLR: 0.7481 (0.6457–0.8505) |
| Blood (Genetic biomarkers) | | | | | |
| Bustos 2020 | USA | cfmiRs | HTG EdgeSeq MiRNA Whole Transcriptome Assay (form of NGS), HTG Molecular Diagnostic Inc, Tucson, Arizona, USA | n.r. | Differently expressed cfmiRs in BM+ vs. HC plasma samples: n=164 (131 were upregulated, 33 were downregulated).  Top 10 most differently expressed cfmiRs: BM+ cohort 1 vs. HC (Log₂(CPM+1): miR-3157-5p: 3.0 (2.5 - 4.0) vs. 2.25 (1.5 - 3.0) miR-339-3p: 3.5 (3.0 - 4.5) vs. 2.0 (1.5 - 3.0) miR-4519: 3.5 (3.0 - 4.5) vs. 2.5 (2.0 - 3.0) miR-4522: 3.0 (2.5 - 4.0) vs. 2.0 (1.5 - 2.5) miR-4664-3p: 3.0 (2.5 - 4.0) vs. 1.5 (1.0 - 2.0) miR-4689: 2.00(1.5 - 2.5) vs. 2.5 (2.0 - 3.0) miR-4706: 3.0 (2.5 - 4.0) vs. 2.0 (1.5 - 2.5) miR-671-5p: 3.25 (2.5 - 4.0) vs. 2.25 (1.5 - 3.0) miR-6852-3p: 3.5 (3.0 - 4.5) vs. 2.00 (1.5 - 2.5) miR-874-3p: 4.0 (3.0 - 5.0) vs. 2.75 (2.0 - 3.5) |
| Faria 2018 | Brazil | cfDNA | fluorimetry (Qubit® Quanti-iT dsDNA BR Assay kit, Invitrogen, São Paulo, Brazil). | MRI | BM+ vs. HC: AUC=0.868, 95%CI: 0.797-0.939 BM+ vs. recurrent glioblastoma: No data provided |
| Blood (Protein) | | | | | |
| Soler 2020 | USA | DVI | 1. DVI calculated by dividing the percentage of circulating HLA-DRneg/low by the percentage of circulating VNN2 cells among CD14 cells 2.CD14+ monocytes were positively selected from PBMC using magnetic CD14 MicroBeads(Miltenyi Biotec, San Diego, California) 3.Mo-MDSC surface biomarkers on CD14+ monocytes were identified using analytic flow cytometry | Pathology | BM+ vs. HC: No data provided  BM+ vs. RN:  DVI : median (range)11.65 (1-63) vs 0.17 (0.05-0.643), p=0.005  HLA- DRneg/low Mo-MDSC:   61.5% vs 7%, p<0.0001  CD14+ VNN2+:  5.5% vs. 62.5%, p=0.0008 |
| Marchi 2008 | USA | S100B Apolipoprotein A1 | ELISA | MRI | S100B (ng/ml): Estimate value from Figure 2 (B1, B2)  BM+ SVID- (n=9) vs. BM- SVID-(n=29): 0.12 vs. 0.27 BM+ SVID+ (n=5) vs. BM- SVID+(n=57): 0.16 vs. 0.22 overall BM+ (n=14) vs. overall BM-(n=86): 0.25 vs. 0.15 |
| Carretero-Gonzalez 2022 | Spain | pSTAT3/STAT3 PD-L1 | Mass spectrometry, proteomic analysis, and immunoblotting Mass spec - Q-Exactive Plus mass spectrometer (ThermoScientific) | CT/MRI | BM- vs. BM+ median (range) estimated from Figure 4 PD-L1 Levels (Relative Units, R.U) Lung: 0.6 (0.1 - 1.8) vs. 0.5 (0.1 - 1.6), p=ns Breast: 0.6 (0.1 - 1.0) vs. 1.2 (0.5 - 4.5),p=ns Kidney: 0.6 (0.1 - 1.8) vs. 0.6 (0.3 - 1.2), p=ns Melanoma: 0.3 (0.1 - 0.6) vs. 3.0 (1.0 - 7.0), p=0.03  pSTAT3/STAT3 Levels (Relative Units, R.U) Lung: 0.4 (0.1 - 1.0) vs. 0.6 (0.1 - 1.8), p=ns Breast: 0.5 (0.2 - 1.5) vs. 1.0 (0.5 - 3.0), p=0.03 Kidney: 0.5 (0.1 - 2.0) vs. 0.3 (0.1 - 1.0), p=ns Melanoma: 1.3 (0.6 - 2.0) vs. 0.1 (0.0 - 0.2), p=0.02 |
| Kim 2021 | South Korea | NFL  GFAP | Simoa HD-1 Analyzer (Quanterix Corporation, Billerica, MA, USA) | MRI | Serum NfL (pg/µL):  Cut-off:  <60 years old: Cut-off = 29.9  60-70 years old: Cut-off = 44.7  >70 years old: Cut-off = 63.9  Median (IQR):  BM+ vs. BM-: 63.7 (29.9–133) vs. 13.3 (10.1–19.9)  BM+ vs. HC: 63.7 (29.9–133) vs. 12.5 (9.8–17.9)  p<0.0001 for both comparison  Sensitivity 91%, specificity 91%  Serum GFAP (pg/mL):  BM+ vs. BM−: 819.5 (321–1650) vs. 154 (121–189)  BM+ vs. HC: 819.5 (321–1650) vs. 135 (90.7–185)  p-value: p < 0.0001 for both comparisons  Sensitivity 91%, specificity 97%  Combined NfL or GFAP:  Sensitivity 98%, specificity 88% |
| Cerebrospinal Fluid (Protein) | | | | | |
| Twijnstra 1987 | Netherlands | LDH | a modification of the enzymatic lactate to pyruvate procedure modified by Gay, McComb and Bowers. | CT CSF cytology histopathology | LDH (U/L): Cut-off: 26 BM+: X=22.6 (SD 28.4), range 0-136,  Control: X=10.2 (SD6.3), range 0-34, p<0.02 |
| Kimura 2018 | Japan | Progranulin (PGRN) | sandwich ELISA | Enhanced brain MRI CSF cytology histopathology | CSF PGRN (pg/dL) Cut-off=2.6  Median (range) carcinoma CNS+ : 4.5 (3.4–9.8) Control 1: 1.0 (0.7–1.1)  AUC: 0.918;  Sensitivity: 90%;  Specificity: 85.1% |

Notes: n.r.=not reported, BM+=brain metastasis, BM-=no evidence of brain metastasis, SVID+ = Subarachnoid vessel involvement (positive), SVID− = Subarachnoid vessel involvement (negative), HC = Healthy control, NT = Nontumorous brain diseases, RN = Radiation necrosis, PCNSL = Primary central nervous system lymphoma, SCNSL = Secondary central nervous system lymphoma, NLR = Neutrophil-to-lymphocyte ratio, dNLR = Derived neutrophil-to-lymphocyte ratio, PLR = Platelet-to-lymphocyte ratio, LMR = Lymphocyte-to-monocyte ratio, PNI = Prognostic nutritional index, SII = Systemic immune-inflammation index, PIV = Pan-immune-inflammation value, cfDNA = Cell-free DNA, cfmiRs = Circulating microRNAs, LDH = Lactate dehydrogenase, PGRN = Progranulin, DVI = HLA-Dr-Vnn2 Index, HLA-DR = Human leukocyte antigen – DR isotype, Mo-MDSC = Myeloid-derived suppressor cells, PD-L1 = Programmed death-ligand 1, pSTAT3 = Phosphorylated signal transducer and activator of transcription 3, OR = Odds ratio, HR = Hazard ratio, AUC = Area under the curve, ELISA = Enzyme-linked immunosorbent assay, CT = Computed tomography, MRI = Magnetic resonance imaging, GC–MS = Gas chromatography-mass spectrometry, LC-qTOF-MS = Liquid chromatography–quadrupole time-of-flight mass spectrometry.

Table S5. Shared biomarkers examined across studies.

| **Biomarkers** | **Biomarker type** | **Tested in studies  (with primary cancer site)** | **Discriminitive ability based on finding from the included studies** | **Conclusion** |
| --- | --- | --- | --- | --- |
| S100B | Protein | Chen 2019 (lung) Choi 2016 (lung) Darlix 2016 (breast) Mu 2017 (lung ) Kondrup 2020 (lung) Marchi 2008 (others not specified) | High diagnostic accuracy in some studies (e.g., Chen 2019, Mu 2017) for lung cancer brain metastases (BM), with consistent elevation in BM+ patients. Variable performance across cohorts and cancer types, with weak diagnostic accuracy for breast cancer (e.g., Darlix 2016) and inconsistent statistical significance in other cohorts (e.g., Kondrup 2020). | Promising for lung cancer BM but limited by variability across settings. |
| Neurofilament light chain (NfL) | Protein | Darlix 2016 (breast) Lin 2022 (lung) Winther-Larsen 2020 (lung) Kim 2021 (colorectal, melanoma, renal and others not specified) | Demonstrated consistent high diagnostic accuracy across multiple cancer types (lung, breast, melanoma), with sensitivity and specificity reaching 91% in Kim 2021. Combined with other biomarkers like GFAP, it showed even greater diagnostic accuracy (e.g., Lin 2022). | Highly effective and consistent biomarker for BM diagnosis across cancer types. |
| Glial fibrillary acidic protein (GFAP) | Protein | Darlix 2016 (breast) Lin 2022 (lung) Kim 2021 (colorectal, melanoma, renal and others not specified) | Shown to perform well individually (e.g., sensitivity 91%, specificity 97% in Kim 2021) and in combination with NfL for enhanced accuracy. Effectiveness varies by cancer type, with weaker performance in lung cancer BM (e.g., Lin 2022). | Strong biomarker when used in multi-marker models; performance varies by cancer type. |
| Anti-S100B IgG | Protein | Chen 2019 (lung) Choi 2016 (lung) Darlix 2016 (breast) | Anti-S100B IgG levels were elevated in BM+ patients in some studies (e.g., Chen 2019), but its diagnostic accuracy was generally weaker compared to S100B alone. Studies like Choi 2016 and Darlix 2016 found it to have limited standalone utility, particularly in breast cancer brain metastases, with low sensitivity and specificity. | Anti-S100B IgG shows limited diagnostic potential as a primary biomarker for brain metastases but may have a role as a supplementary marker in combination with other biomarkers like S100B. Further validation in larger cohorts and across different cancer types is needed to establish its clinical relevance. |
| Carcinoembryonic antigen (CEA) | Protein | Cacho-Diaz 2019 (lung) Darlix 2016 (breast) Lee 2012 (lung) | Found to have moderate diagnostic accuracy in lung and breast cancer BM (e.g., AUC values between 0.6–0.7 in Cacho-Diaz 2019 and Lee 2012). Results are inconsistent, with thresholds varying across studies. | Limited standalone diagnostic utility; potential when used in combination. |
| Neutrophil-to-lymphocyte ratio (NLR) | Cell/cellular component | Rojko 2020 (lung) Sert 2021 (lung) Yang 2023 (others not speicified) | NLR showed promise as a biomarker in Sert 2021, Rojiko 2020 and Yang 2023, with significant differences observed between BM+ and BM− groups (Rojko 2020, Yang 2023). However, variations in cutoff values and patient populations affected consistency. | NLR has moderate potential as a diagnostic biomarker for brain metastases but requires standardization of thresholds across studies. |
| Platelet-to-lymphocyte ratio (PLR) | Cell/cellular component | Rojko 2020 (lung) Sert 2021 (lung) Yang 2023 (others not speicified) | PLR demonstrated some diagnostic potential in Sert 2021. However, no evidence of its effectiveness was found in Rojko 2020 and Yang 2023, its effectiveness varied across cancer types. | PLR is a less effective biomarker for brain metastases but may be more useful in combination with other inflammatory markers. |
| Neuron Specific Enolase (NSE) | Protein | Darlix 2016 (breast) Li 2016 (lung) | NSE was evaluated in only two studies, showing moderate diagnostic potential for brain metastases. However, its sensitivity and specificity varied, limiting its standalone diagnostic utility. | NSE may have some diagnostic value, but its effectiveness is inconsistent and likely limited without combination with other biomarkers. |
| Lactate dehydrogenase(LDH) | Protein | Sert 2021 (lung) Twijnstra 1987 (others not specified) | LDH levels were elevated in cerebrospinal fluid (CSF) of brain metastasis-positive patients in studies such as Twijnstra 1987, with diagnostic potential reported in certain cancer types. However, variability in thresholds across studies limits its broad applicability. | LDH is a promising CSF biomarker for brain metastases detection but requires standardization and further validation. |
| Platelet count (PLT) | Cell/cellular component | Rojko 2020 (lung) Sert 2021 (lung) | PLT differences between BM+ and BM− patients were observed in two studies , but its diagnostic utility remains unclear due to overlapping ranges in healthy and diseased populations. | PLT alone is unlikely to be an effective biomarker for brain metastases and requires further evaluation in combination with other markers. |
| cfDNA | Genetic biomarker | Augus 2021 (breast) Faria 2018 (breast, lung, pancreas, colon, prostate) | cfDNA showed high diagnostic potential in Faria 2018 and Angus 2021, with elevated levels distinguishing BM+ from BM− patients. Its performance was particularly strong in cerebrospinal fluid-based detection. | cfDNA is a promising biomarker for brain metastases, particularly when sampled from CSF. Further validation in larger and diverse cohorts is needed. |
